# Supplementary material for: Flat-Top Line-Shaped Beam Shaping and System Design
Source: Sensors (Basel). 2022 May 31;22(11):4199. doi: 10.3390/s22114199 (PMC9185535; doi:10.3390/s22114199)
Supplement: Supplementary file 1 [file sensors-22-04199-s001.zip › sensors-1739637-supplementary.pdf]

The geometrical dimensions of aspherical cylindrical lens 1, aspherical cylindrical lens 2, cylindrical lens 3 and cylindrical lens 4 are shown in Figures S1–S4.

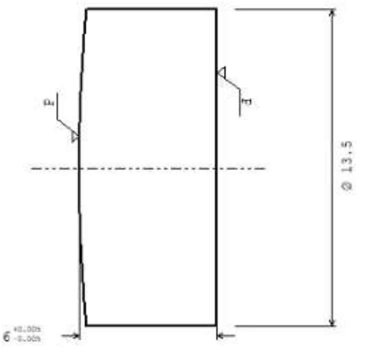

Dimensions in Millimeters

Left Surface	Material	Right Surface
R 64.5522 $^{+0.001}_{-0.001}$ CX	GLASS: ZNSE	R PLANO
Toroidal	N(10.5 µm) = 2.403309 $^{+0.001}_{-0.001}$	$Ø_K$ 13.5
See Attachment	V -	⊘
K -64.6716 $^{-64.66}_{-64.68}$	0/ -	3/ 1.0(0.02) @ 10.6 µm
$Ø_K$ 13.5	1/ -	4/ 0.6' (0.005)
⊘	2/ -	5/ -
3/ @ 10.6 µm		6/ -
4/ -		

Figure S1. Geometrical dimensions of aspherical cylindrical lens 1

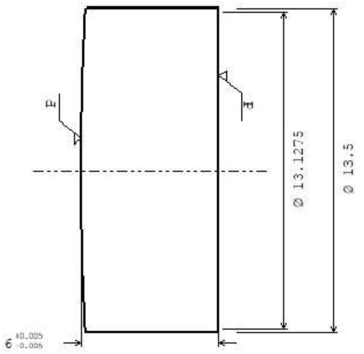

Dimensions in Millimeters

Left Surface	Material	Right Surface
R 123.491 $^{+0.001}_{-0.001}$ CX	GLASS: ZNSE	R PLANO
Toroidal	N(10.5 µm) = 2.403309 $^{+0.001}_{-0.001}$	$Ø_K$ 13.1275
See Attachment	V -	⊘
K -85.9817 $^{-85.97}_{-85.99}$	0/ -	3/ 1.0(0.02) @ 10.6 µm
$Ø_K$ 13.5	1/ -	4/ 0.6' (0.005)
⊘	2/ -	5/ -

Figure S2. Geometrical dimensions of aspherical cylindrical lens 2

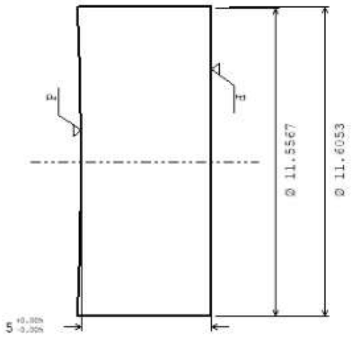

Dimensions in Millimeters

Left Surface	Material	Right Surface
R 124.613 $^{+0.001}_{-0.001}$ CC	GLASS: ZNSE	R PLANO
Toroidal	N(10.5 μm) = 2.403309 $^{+0.001}_{-0.001}$	Øₑ 11.5567
See Attachment	V -	Ø
Øₑ 11.6053	0/ -	3/ 1.0(0.02) @ 10.6 μm
Ø	1/ -	4/ 0.6' (0.005)
3/ @ 10.6 μm	2/ -	5/ -
4/ -		6/ -
5/ -		

Figure S3. Geometrical dimensions of cylindrical lens 3

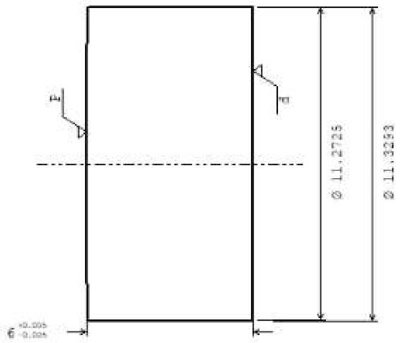

Dimensions in Millimeters

Left Surface	Material	Right Surface
R 266.913 $^{+0.001}_{-0.001}$ CX	GLASS: ZNSE	R PLANO
Toroidal	N(10.5 μm) = 2.403309 $^{+0.001}_{-0.001}$	Ø 11.2725
See Attachment	V -	Ø
Ø 11.3293	0/ -	3/ 1.0(0.02) @ 10.6 μm
Ø	1/ -	4/ 0.6' (0.005)
3/ @ 10.6 μm	2/ -	5/ -
4/ -		6/ -

Figure S4. Geometrical dimensions of cylindrical lens 4
